# Supplementary material for: Berberine Promotes Beige Adipogenic Signatures of 3T3-L1 Cells by Regulating Post-transcriptional Events
Source: Cells. 2019 Jun 23;8(6):632. doi: 10.3390/cells8060632 (PMC6627823; doi:10.3390/cells8060632)
Supplement: Supplementary file 1 [file cells-08-00632-s001.pdf]

Berberine promotes beige adipogenic signatures of 3T3-L1 cells by regulating post-transcriptional events

Ying-Chin Lin[1], Yuan-Chii Lee[2], Ying-Ju Lin[3,] Jung-Chun Lin[4,5,*]

[1]Department of Family Medicine, Shuang Ho Hospital, Taipei Medical University, New Taipei City, Taiwan; greening1990@gmail.com (Y.-C.L.))

[2]Graduate Institute of Biomedical Informatics, Taipei Medical University, Taipei, Taiwan; ycgl@tmu.edu.tw (Y.-C.L.)

[3]School of Chinese Medicine, China Medical University, Taichung, Taiwan; yjlin@mail.cmu.edu.tw (Y.-J.L.)

[4]School of Medical Laboratory Science and Biotechnology, College of Medical Science and Technology, Taipei Medical University, Taipei, Taiwan; lin2511@tmu.edu.tw (J.-C.L.)

[5]PhD Program in Medicine Biotechnology, College of Medical Science and Technology, Taipei Medical University, Taipei, Taiwan ; lin2511@tmu.edu.tw (J.-C.L.)

***Correspondence: lin2511@tmu.edu.tw; Tel.: +886-2-27361661 (ext. 3330)

Fig. S1

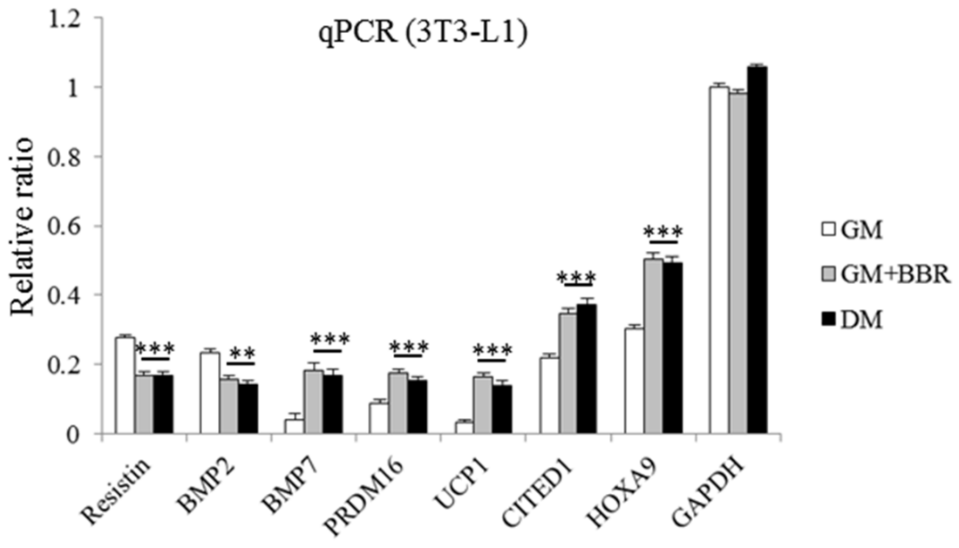

Supplementary Figure S1. Berberine (BER) treatment modulates the adipocyte-related gene expressions. 3T3-L1 cells were cultured in growth medium, BBR-supplemented growth medium (5 μM) or differentiation medium for 48 h. Total RNAs extracted from the *in vitro* cultured cells were subjected to qPCR assays using specific primer sets as listed in supplementary Table 2.

Supplementary Table S1. PCR Primers.

Target	Forward	Reverse
LIPIN1-AS	tcccagttcggacagagaat	gccagagcatttccaggtta
PKM-AS	agaaacagccaaaggggact	caacatccatggccaagtt
PRDM16-AS	gagccccaaggagtctatga	cgagggtcctgtgatgttcaa
BMP2	ccaagagacatgtgaggatt	ttagtggagttcaggtggtc
Resistin	aagaacctttcatttcccctcct	gtccagcaatttaagccaatgtt
CITED1	cggggtcaccgcaaatgga	ctcatccaccgggtcagaa
HOXA9	cagtgtatcatcaccaccacca	gaggagaaccacaagcatagt
PRDM16-Total	gtcagaggagaaatttgatgg	agaaggaatgctgtgagtag
UCP1	ctcaggattggcctctacgactc	ttggtgtacatggacatcgca
RBM4a	taggatccgcagtgcgtac	ttaaaaggctgagtccgcg
SRPK1	tccaggcccgaaagaaa	gaatatcccatgataacca
GAPDH	cggagtcaacggatttggtcgtatg	agccttctccatggtggtgaagac
SP1	tctgctctccatcctatgtt	tagggaaagaccactgagg
MYC	gtgaacttgacctcacagc	tggaactgtgggattacttg
C13orf25 (promoter)	atatagatcttgccgccgggaaacgggtt	atataagcttccatacaaattcagcataatcc
miR-92a	tattgcacttgtcccggcctgt	ccgaggcggccgacatgttt
miR-485	agaggctggccgtgatgaattc	ccgaggcggccgacatgttt
miR-92a expressing	gtgaattcctgtgtgatattctgctg	ggctcgagtgccaaatctgacacgc
RBM4a (201-652)	gtgaattcctgtgtgatattctgctg	atcgcggccgcgcgctccgtacgcgttgttg

Supplementary Table S2. qPCR Primers.

Target	Forward	Reverse
BMP2	ccaagagacatgtgaggatt	ttagtggagttcaggtggtc
Resistin	cctacacactcagcagtacggac	tcaaactctgtatagaacttgcgga
CITED1	cggggtcaccgcaaatgga	ctcatccaccgggtcagaa
HOXA9	cagtgtatcatcaccaccacca	gaggagaaccacaagcatagt
PRDM16	gacattccaatcccaccaga	cacctctgtatccgtcagca
UCP1	tacacggggacctacaatgct	ggtcactggaagatatggc
RBM4a	atgccttcgtacacatggagcg	ggctggtggacaactgcacgt
SRPK1	cctcattcaggggaagagtaca	ctgccacaatgagcttgc
GAPDH	aaggtcatcccagagctgaa	ctgcttcaccaccttcttga
miR-92a	tattgcacttgtcccggcctgt	ccgaggcggccgacatgttt
miR-485	agaggctggccgtgatgaattc	ccgaggcggccgacatgttt
